# Integrative Approaches to the Undergraduate Public Health Major Curriculum: Strengths, Challenges, and Examples

**DOI:** 10.3389/fpubh.2019.00106

**Published:** 2019-04-30

**Authors:** Marc T. Kiviniemi, Sarahmona M. Przybyla

**Affiliations:** ^1^Department of Health, Behavior and Society, College of Public Health, University of Kentucky, Lexington, KY, United States; ^2^Department of Community Health and Health Behavior, School of Public Health and Health Professions, University at Buffalo, Buffalo, NY, United States

**Keywords:** undergraduate public health, BSPH, integrative public health curriculum, curriculum designed, student learning

## Abstract

Many “first generation” undergraduate public health degree programs were designed based on “siloed” course structures centered around subunits in the discipline (e.g., Introduction to Epidemiology, Introduction to Environmental Health) that may be meaningful primarily to experts in the field. An alternative to the siloed approach is an integrative curricular design, in which courses are designed around meaningful thematic units (e.g., explaining public health problems, asking and answering scientific questions in public health), with an emphasis on drawing connections between knowledge from different but complementary disciplinary areas as a means to improve student learning and retention. The integrative approach shifts the curriculum conversation to capitalize on the interdisciplinary roots of the public health profession. This approach is consistent with the learning outcome recommendations in the Framing the Future Task Force report and in the CEPH requirements for the undergraduate public health major. We explore integrative approaches to developing curricular models for undergraduate public health programs and discuss both pedagogical and career preparation arguments supporting an integrative curriculum approach. These include facilitating the often-challenging task for students of seeing how concepts interrelate, making transparent how “basic” knowledge in the discipline relates to “real world” applications of the content, and better mirroring how professionals in the discipline actually use knowledge in practice. Finally, we review examples of core concepts and features in an integrative curriculum approach to the undergraduate public health major as an effective educational program with high-quality, learner-centered educational experiences.

## Introduction

The goal of any undergraduate curriculum, including public health, we would argue, is to accomplish two objectives. First, it should teach students the “habits of thought” that characterize the discipline—how do public health professionals think about problems, analyze them, and go about solving them? Second, it should equip students with the essential skills necessary to function as entry-level public health professionals [for discussion of these two goals and their possible rapprochement see (1)].

As a way of considering what is involved in meeting these two goals, consider a classic problem in public health education—the “potato salad” problem (2). A group of people goes on a picnic and they eat several foods, including potato salad. Later, some of them get sick (and, in true public health fashion, some of them do not). They provide some data on what foods they ate at the picnic, and the task for our budding public health professional is to decide whether they got sick *because* they ate the potato salad. As it has been traditionally done in class, this is epidemiology content pure and simple—students craft out a table calculating the disease rate for those who ate the potato salad relative to those who did not and, if they want to get really fancy, compare that to the disease rate for people who ate other foods vs. those who did not. A few simple calculations later and, voilà, the potato salad is identified as the cause of the sickness and students have a piece of knowledge on how to use an epidemiological principle to answer a public health question.

As a class example to teach and test a piece of knowledge from epidemiology, this works well as is. But going back to the articulated goals for a public health curriculum, we need to consider phenomena as they would occur in the “real world.” In the real world of public health, the problem that would present itself to a member of the public health workforce wouldn't be friends on a picnic, it would be multiple people in the community buying vats of potato salad at the grocery store's deli counter to serve at their picnics and a set of ambiguous, incomplete case reports trickling in from around the community and needing to be assembled into a coherent whole. Also, in the real world a data table wouldn't magically appear from which students could calculate disease rates. The public health worker would have to use community engagement skills to go out, collect relevant data, assemble information from people's memory, and overcome community suspicions. Then, the data analytic skills above would surface. Even then, though, the real world work wouldn't be done. The public health worker would have to consult with a legal team to decide what steps could be taken to address the situation within the bounds of the city or county health department's authority. Health communications work would then need to come into play to develop the optimal strategy for getting the information disseminated to the public in a way that not only informs but also leads to needed action. The contrast between the class example and the real world is telling. Real world public health requires the integration of knowledge and skills from across the spectrum of traditional public health disciplines in order to understand and address the problem. It also requires a more complex set of thinking and analytic skills than simply the calculation of an odds ratio from a 2 × 2 table.

The primary question for public health education, and the question we seek to address in this paper is: how do we best define and develop undergraduate public health education programming in a way that allows us to simultaneously meet two critical goals—to both best educate students to understand and analyze the complexities involved and to ensure that students leave our programs with skills that allow them to do things to address these complex problems when they engage in the “real world” work of public health practice? This real world of public health involves the kinds of complexities in the examples above, and such complexities are growing over time as we shift toward a public health paradigm that explicitly addresses social determinants (3), pushes for an approach of health in all policies (4), and shifts focus toward one health (5) and other explicitly interdisciplinary, multilevel approaches. In doing so, we ask a key question at the level of the curriculum for the undergraduate public health major—what high impact, learner-center practices and approaches should be used in developing and implementing a curriculum in order to maximize student learning and student outcomes?

In this paper, we make the argument that achieving the goals of undergraduate education within the interdisciplinary context of public health is best done by designing curricula using an intentional, integrative approach. We begin by discussing what is meant by an integrative approach. We then advance arguments for the effectiveness of an integrative approach within the context of the goals of undergraduate education and why we would characterize it as a high impact approach to the undergraduate public health curriculum. Finally, we describe an exemplar of an undergraduate public health (UGPH) curriculum developed using principles of integrative curriculum design.

## What is an Integrative Curriculum Model?

To develop an integrative Bachelor of Science in Public Health (BSPH) curriculum, one's team must first examine existing definitions. Jacobs defines interdisciplinary learning as “a knowledge view and curriculum approach that consciously applies methodology and language from more than one discipline to examine a central theme, issue, problem, topic, or experience” [(6), p. 8]. Public health curriculum developers must also recognize the discipline field as a “specific body of teachable knowledge with its own background of education, training, procedures, methods, and content areas” [(6), p. 7].

The key feature of an integrative curriculum model is taking a conscious approach to integrating in coursework and drawing explicit educational connections between pieces of knowledge from particular separate disciplinary backgrounds (7). Thus, one does not define the curriculum in terms of a set of courses that individually cover particular disciplinary domains, but rather defines in terms of common questions, problems, ideas, skills or other meaningful thematic units built from the connections drawn as described above. Consequently, integrative curricula can have a variety of definitions [for examples see (8, 9)]. A common approach in medical education has been to replace separate and distinct “disciplinary” courses in the first 2 years of the curriculum with interdisciplinary courses organized around organ systems (10). In our UGPH program, the organization of a curriculum around meaningful thematic units was designed with both attention to what thematic organization best meets student learning objectives and best captures the “real world” uses of the curriculum material.

## Why an Integrative Curriculum Approach?

With the definition of an integrative curriculum model in mind, we now turn to a brief discussion of why a unit or department would want to consider an integrative approach to developing an undergraduate public health curriculum and the advantages that it may have over the more traditional, siloed approach. Why would one design or redesign an undergraduate public health curriculum from an integrative standpoint?

### What Are the Goals of an Undergraduate Curriculum?

Central to our arguments about the advantages of an integrative approach relative to the traditional, siloed model is a set of basic assumptions about the desired outcomes of an undergraduate degree in public health. We assume that any undergraduate public health program has as its goal meeting at least four key outcomes.

First, we want to develop and implement a curriculum that prepares undergraduate students with the knowledge, skills, and values necessary to enter the public health workforce (broadly defined) ready to successfully begin their careers or further their education and contribute to addressing the public health needs of the population from their first jobs and continuing through the remainder of their careers (11, 12).

Second, we want to offer a curriculum that introduces students to how public health thinks about the world, the ideas and lenses through which one takes a public health perspective in explaining and preparing to change and improve the world, that provides reasoning and problem-solving skills and that introduces students to the values and underlying principles that inform the public health perspective (1). Although programs and curricula can vary in the relative weighting they place on goals one and two (13) both are, we would argue, necessary for a high-quality curricular approach.

Third, for undergraduate programs situated within schools or programs of public health or for those that have pursued standalone baccalaureate program status from the Council on Education in Public Health, a key goal is to ensure that the curriculum allows full satisfaction of the required learning outcomes for an accredited undergraduate degree in public health. The accreditation requirements for schools and programs offering the undergraduate degree articulate both content knowledge (e.g., “the socioeconomic, behavioral, biological, environmental, and other factors that impact human health and contribute to health disparities”) and demonstrable skills (e.g., “the ability to locate, use, evaluate and synthesize public health information”) that must be addressed in the curriculum (14).

The preceding goals don't occur unless we achieve a fourth, final goal—a goal of undergraduate education (and truthfully, all higher education), is to produce student learning and especially to produce high-quality learning that a student retains over a period of time—in other words, although the task we undertake in our classrooms is providing instruction, the ultimate goal in our courses and overall programs is to *produce learning* in our students (15, 16). Although this seems like a statement of the blindingly obvious, it needs to be explicitly stated because designing the curriculum in a way that best produces long-term learning needs to be an explicit goal when one engages in curriculum design.

### How and Why Does an Integrative Curriculum Help With Meeting These Goals?

So, from the viewpoint of these four goals for undergraduate education, why is an integrative curriculum approach potentially a better bet than the traditional, siloed approach? Let's consider potential integrative curriculum advantages from the vantage point of the four goals of undergraduate education outlined above.

#### The Nature of the Field of Public Health

For the first goal, preparing students for the workforce, the core argument for the integrative curriculum approach is that the work that is done in public health is *inherently* integrated. Very few of the tasks a public health professional undertakes in her daily life, and virtually none of the problems that public health professionals tackle, neatly fit into a single one of the silos defining the traditional curriculum approach.

The potato salad example above illustrates this point nicely. Identifying, developing plans to address, intervening to address, and evaluating whether the intervention was effective involves knowledge and skills that span multiple knowledge domains within public health. Virtually every public health example is similar. A group of residents from a particular neighborhood are concerned about what they perceive as high rates of respiratory problems in their children. Is something going on and if so, how is the local health department going to respond? Even answering the “is there a disease cluster?” in this community question involves the combined knowledge set of epidemiology and biostatistics, and the skills to gather the requisite exposure information from community members and health outcome knowledge requires skills from both community health and health services perspectives. We're only one step into fully addressing the problem and already addressing it involves skills in four of the traditional knowledge domains of public health.

We leave it to the reader to conduct additional thought experiments here if additional illustrations are needed. Suffice it to say that we take as a given that “real world” public health work has an inherent and inescapable requirement for integrated knowledge and skills across public health disciplines. We also take it as a given that as the public health challenges faced by the population shift over time, the need for integrated knowledge and skills will only grow. Emerging perspectives shaping the field all require more integrated knowledge and skills than did John Snow's work with the Broad Street Pump in 1854 (17) (and we would argue that there was some integrated knowledge at work even there).

A core principle in education and curriculum design is that one provides a learner with mastery of a set of program-level learning outcomes by providing instruction, practice, and the ability to demonstrate mastery of the types of skills that the learner will need to perform to be successful moving forward. To that end, if the reader buys our argument that the “real world” skills our undergraduate public health students will be expected to perform are integrated skills, then the work we do in our classrooms must be to teach and allow the opportunity to master those skills. If we teach a series of siloed courses in which students learn and practice skills in epidemiology in an epidemiology course and skills in biostatistics in a biostatistics course and so on, when and where do they get the opportunity to practice the integration of those skills?

In a siloed curriculum model, the student is most often left to master the integration of skills on her or his own. This approach rests on two rather tenuous assumptions–that the student *should* master them and that the student *can* master them. The *should* part comes down to a belief about educational philosophy. While we hope that the reader will be convinced by the end of this paper that students *should not* be left to master integration of siloed content knowledge on their own, that has to be a judgment made by the curriculum designers.

The question of whether students *can* master siloed knowledge integration on their own is an evidence-based question. The predominance of evidence suggests that the answer to this question is more often than not no. Evidence on retention of knowledge and ability to integrate skills across domains shows that often students are unable to integrate unaided (18, 19). Although integrating information and applying it to novel situations is a key educational outcome, students often do not integrate and extend application successfully on their own [e.g., (19)]. In fact, some attempts to accomplish integration by maintaining siloed courses but using common themes and topics to draw connections have found that students didn't even notice the integrated material until it was explicitly pointed out by faculty members.

#### The Nature of Student Learning

As discussed above, the central goal of education is not teaching a particular set of content; it is creating student learning, particularly learning that is long-lasting and able to be effectively retrieved and used when needed in a student's post-collegiate life and work (15, 16). Curriculum designs focused on ideas, themes, and answering public health questions can be more effective than designs focused on delivering information and leaving it to students to integrate (16, 20, 21). As such, improved learning outcomes and improved student motivation are core arguments for the integrative approach (22–24).

Public health is an evidence-based discipline–we (rightly) ask about the evidence base for interventions and policy approaches to address public health problems, we recognize assessment and evaluation as core functions of public health, and at all levels of the curriculum, we take as a core goal providing students the skills to assess, interpret, and evaluate the evidence for an approach. It behooves us, then, to apply evidence-based practices to the design of our curricula and courses. There are a variety of articulations of these best practices [e.g., (25)] and, notably, siloed curriculum approaches are not among them. Indeed, an overview of teaching best practices for long-term learning argued that “…it would be difficult to design an educational model that is more at odds with current research on human cognition than the one that is used in most colleges and universities” [(25), p. 4]. Within the context of these best practices, integrative learning is frequently cited as a high-quality educational practice and as a means of producing high-quality, long-lasting learning. Integrating allows focus on the “big ideas”—often cited as a best practice in curriculum design (16). The American Association of Colleges and Universities (AACU) has argued that the ability to integrate and synthesize knowledge is one of the four essential outcomes of an undergraduate educational experience (26).

Accomplishing integrative learning involves several shifts—from “surface level” taking in of facts to “deeper level” thinking about connections across time and across contexts (27), from thinking about disciplinary and course contexts as isolated from one another to thinking about the webs of connection that tie things together to solve “real world” issues (19).

Unfortunately, typical undergraduate students are unlikely to learn very real and necessary connections on their own. There are two types of integration involved—first, students may not see how concepts from one course/discipline/module connect to related concepts from another course/discipline/module (typically termed horizontal integration). Second, students may not see how “basic” or “conceptual” material concepts relate to applications and professional lives (vertical integration) (10). Noticing, appreciating, and accomplishing integration is often one of the most challenging cognitive tasks we ask students to undertake. By making explicit the connections between ideas, and indeed framing the curriculum around those connections, the integrative curriculum approach addresses directly the difficult cognitive task that, in more traditional siloed approaches, is left to students to accomplish on their own.

In addition to the direct cognitive task of integrating knowledge, a second learning-based argument for the integrative approach is that learning is only effective if it is long-term learning that can be retrieved and used in the future. In terms of teaching and learning best practices, the argument has been made that “We need to provide an education that lasts a lifetime, which means thinking beyond the end of the semester, and let the learning principles for long-term retention and flexible recall guide our teaching practices” [(25), p. 4].

In the context of long-term retention and retrieval of information, some key principles of human memory and how humans learn highlight advantages of the integrative approach. In order to be remembered, retrieved, and used, delivered course content has to be tied to other, pre-existing memory structures (28). An item in isolation is much less likely to be effectively learned and remembered than one that is tied to pre-existing knowledge. Organization and association of information in memory is key to long term retention and undemanding. It aids initial understanding by contextualizing the information in light of other information, but also aids the likelihood of long-term recall (29, 30). Full mastery of learning content involves multiple steps—acquiring skills/knowledge; integrating the individual skills and knowledge with others; and then applying those skills and knowledge appropriately to address and understand problems, including extending the domains for that application past the specific domain in which the skill was acquired (18).

Finally, not only can integrative curriculum designs aid students in seeing and understanding critical interconnections between content domains and in learning course material in ways conducive to long-term information retrieval, such design also aids in developing critical higher-order thinking and reasoning skills. One example of this is creative generation of new ideas. One conceptual approaching and fostering creativity is a “Janusian approach” in which creative thinking involves creating connection between ideas that might otherwise not seem connected at all (31). To the extent that we want our students to be both creative thinkers and problem solvers, arguably a core professional skill for public health where problem solving and intervention development involves working within systems and with multiple constraints to solve health problems that may not have existed when the student was in college, developing the ability to see connections between seemingly disparate ideas is critical. As discussed above, most students are unlikely to see or form such connections on their own and therefore a goal of education has to be teaching those connections as a core, concrete skill to be introduced practiced, and mastered in the course of the undergraduate program (7).

As another thinking skills domain, consider the need to take ideas and skills developed in the classroom and apply those skills to solving a problem that may be in a novel domain. Such an ability to transfer across domains is not inherent or automatic and therefore must be learned, and curriculum design needs to take this necessary learning into account. We know that repeated practice with different applications and different spheres is advantageous—organizing around the skill/topic may help (32). In fact, some have even argued that course content should be framed by laypeople because laypeople tend to think about things in terms of actual problems to be solved whereas professors tend to focus on subject matter (33).

For all of these student learning-focused reasons, we argue that an integrative curriculum approach offers advantages in engaging student learning. We are not the only ones to support this strategy. Many curriculum reform efforts advocate for integrative learning as a core feature of high-quality educational programs across various disciplines? (20, 34, 35). The AACU argues for creating “intentional learners” and defines that, among other things, “Intentional learners are integrative thinkers who can see connections in seemingly disparate information and draw on a wide range of knowledge to make decisions. They adapt the skills learned in one situation to problems encountered in another” [(36), p. 21].

In the context of undergraduate degree programs in public health, where there are accreditation requirements specified by the Council on Education in Public Health, one can also consider the learning advantages of the integrative curriculum approach from the perspective of meeting accreditation requirements. Although the CEPH accreditation requirements (14) do not specify any particular curricular approach, we would argue that they do implicitly articulate a vision for undergraduate public health education that has integrative themes woven throughout. Themes like multilevel, social-ecological approaches to understanding and addressing problems, systems thinking, and synthesis of information are all inherently integrative. Moreover, regardless of the curricular approach taken to address the knowledge and skill domains specified by CEPH, one must demonstrate student learning and outcomes relevant to those domains. To that extent, the articulated advantages of the integrative approach for student learning are also advantageous for satisfying the accreditation requirements of ensuring student learning of what is inherently an integrative, interdisciplinary field of study.

#### Curriculum Synergies

As a final argument for the advantages of an integrative undergraduate public health curriculum, our experience of developing the integrative curriculum approach described in detail below is that there were interesting, unexpected synergies that occurred when considering the curriculum from an integrative perspective. The first of those synergies is that we discovered there were some topics that don't have a natural “home” in any of the siloed, disciplinary courses typical of non-integrated curricula but that emerge and fit quite nicely in an integrative approach. For example, our integrative curriculum includes a course on public health intervention approaches that uses the social-ecological model as an organizing framework. When developing content for the course, we realized that food fortification was an ideal public health strategy to talk about as a basic biological intervention in the ecological framework. We then realized that food fortification wouldn't likely come up naturally in any of the coverage of intervention approaches in a siloed model—health education and individual interventions would be covered in health behavior, policy approaches would be addressed in a health systems/services course, and screening would be covered in epidemiology, but food fortification wouldn't fit neatly into any of the necessary disciplinary “bins” and therefore likely wouldn't be covered.

In addition, the integrative approach allows for efficiencies in coverage of key concepts that are more challenging to achieve in a siloed approach. Consider the issue of different models for explaining public health problems. We use the epidemiologic triad to explain infectious disease transmission, the exposure pathways model to describe human exposure to environmental pollutants, and the social-ecological model to describe the complex causation of chronic disease risk (and other public health outcomes). Understanding each of the models involves being able to think about multiple constructs as causes or influences, characterizing and understanding the interrelations between the multiple constructs, and understanding the different ways in which constructs can intersect and interact to determine outcomes.

In a siloed approach, where the epidemiologic triad would be covered in an epidemiology course, the social-ecological model in a health behavior course, and the exposure pathways model in an environmental health course, the relevant tasks of understanding multiple causes would have to be taught each time. In an integrative curriculum model, though, one can organize an undergraduate course around ways of understanding and explaining public health problems, teach the basic logic of multiple causes early on in the semester, and then introduce all three models in turn and use teaching and applying them as a way to provide repetition and practice across novel contexts as each model is learned and the principles of multiple causation observed and used repeatedly.

## An Example Integrative Curriculum Approach

At the University at Buffalo's School of Public Health and Health Professions, the UGPH program began in Fall 2017 as the first ever BSPH program in the School. Incoming freshmen began direct admission in Fall 2018. From its inception, the UGPH curriculum is designed with five key elements: (1) major building blocks, (2) introductory coursework, (3) upper-level coursework, (4) electives, and (5) one capstone course. The major building block courses entail 11 credit hours in the College of Arts and Sciences and include coursework in chemistry, political science, and statistics as well as 4 credit hours in the School of Medicine for a human physiology course. At the introductory level, students take two required lower division courses, with 200–300 students typically in each course. These courses expose them to a broad overview of the discipline and include basic principles of population health, with an integration of both historical and contemporary public health problems as a method to improve public health via the explicit integration of both content knowledge and approaches from all five core areas. At the upper division level, students take 16 credits over five courses, each currently capped at 75 students. Each integrate core curriculum content in a reflective manner, where students are challenged to assimilate the subject matter using deliberate teaching and evaluation criteria. At the upper division level, students complete nine credits of upper-level electives from a growing menu of options (e.g., Public Health Nutrition, Social Determinants of Health). Finally, a four-credit capstone experience offers students the opportunity to synthesize and apply the knowledge and skills developed in previous coursework and out-of-classroom experiences in a holistic way. The capstone is capped at 30 students with the first cohort of BSPH graduates in Spring 2019. We envision several flavors of the capstone including but not limited to an independent research project, a study abroad experience, and a public health internship at a partnering public health agency (see Tables 1, 2). The program currently has approximately 280 students enrolled.

**Table 1:** Linkages between critical components elements, domains, and courses in the Undergraduate Public Health Major at the University at Buffalo.

**Critical components elements**	**Domain**	**Course number and title**
Background domains	Science	CHE 101: General chemistry PGY 300: Human physiology
	Social and behavioral sciences	PSC 101: Introduction to american politics PSY 101: Introductory psychology SOC 101: Introduction to sociology
	Math/quantitative reasoning	STA 119: Statistical methods
	Humanities/fine arts	ENG 285: Writing in the health sciences
Public health domains	Overview of public health	PUB 101: Introduction to public health PUB 102: Historical and contemporary public health problems
	Role and importance of data in public health	PUB 315: Asking and answering scientific questions in public health
	Identifying and addressing population health challenges	PUB 101: Introduction to public health PUB 210: Global public health PUB 220: Behavioral and social influences on health
	Human health	PUB 310: Health and disease: biological, personal, and environmental influences
	Determinants of health	PUB 101: Introduction to public health PUB 210: Global public health PUB 220: Behavioral and social influences on health
	Project implementation	PUB 325: Interventions to address public health problems
	Overview of the health system	PUB 330: Public health systems and policies
	Health policy, law, ethics, and economics	PUB 102: Historical and contemporary public health problems PUB 330: Public health systems and policies PUB 422: Public health ethics: an interdisciplinary exploration
	Health communication	PUB 320: Models and mechanisms for understanding public health PUB 325: Interventions to address public health problems PUB 494: Capstone: modern public health problems and solutions
Cumulative experience and field experience	Cumulative experience: cumulative, integrative, and scholarly or applied experience or inquiry project that serves as a capstone to their educational experience	PUB 494: Capstone: modern public health problems and solutions
Cross-cutting areas	Advocacy for protection and promotion of the public's health at all levels of society Community dynamics Critical thinking and creativity Cultural contexts in which public health professionals work Ethical decision making as related to the self and society Independent work and a personal work ethic Networking Organizational dynamics Professionalism Research methods Systems thinking Teamwork and leadership	PUB 101: Introduction to public health PUB 102: Historical and contemporary public health problems PUB 310: Health and disease: biological, personal, and environmental influences PUB 315: Asking and answering scientific questions in public health PUB 320: Models and mechanisms for understanding public health PUB 325: Interventions to address public health problems PUB 330: Public health systems and policies PUB 494: Capstone: modern public health problems and solutions

**Table 2:** Undergraduate public health courses at the University at Buffalo.

**Course number and title**	**Course description**
PUB 101: Introduction to public health	The course is designed to provide you with an understanding of and appreciation for population approaches to improving the health of our nation and the world, as well as knowledge of various career paths in public health.
PUB 102: Historical and contemporary public health problems	This course is an integrative overview of both historical and contemporary public health problems and how they were/are being addressed. The course also introduces students to the public health approach to improving health by integrating approaches from the five core areas of the discipline.
PUB 210: Global public health	This course will provide upper division undergraduate students with a meaningful appreciation of the challenges in achieving the human right to health in low- and middle-income countries worldwide. Students will understand the leading causes of illness, death, and disability and approaches to prevention and control of those conditions in resource-constrained settings. Students will also understand the complex interrelationships between social, environmental, and political factors that affect health and well-being in low- and middle-income countries.
PUB 220: Behavioral and social influences on health	The discipline of public health helps inform decisions that shape the behavior of individuals, communities, and societies. PUB 220 is an exploration of theories, models, and methods of social and behavioral disciplines relevant to the identification, description, and solution of public health problems. The course is designed to engage students' curiosity and aid them in developing basic literacy as well as critical and creative thinking regarding social and behavioral concepts and processes that influence personal and population health.
PUB 310: Health and disease: biological, personal, and environmental influences	This course provides an overview of the biological bases of health and illness as well as an overview of the intersections of biological, personal, and environmental determinants of health and illness. Students will learn about key biological processes and physiological systems relevant to public health issues as well as how biology and the environment interact to lead to health outcomes.
PUB 315: Asking and answering scientific questions in public health	This course provides an overview of scientific methodology and evidence-based practice in public health. Students will learn about the research methods used to collect data and the statistical methods used to evaluate that data in public health research and practice. Students will also gain exposure to how those methods are used to address problems in public health.
PUB 320: Models and mechanisms for understanding public health	This course addresses how we understand and explain the causes of public health problems. Students will gain an understanding of the complex causes of different types of public health problems, including infectious diseases, chronic diseases, and environmental health hazards. A particular focus will be on how the person and the environment interact to influence health and illness.
PUB 325: Interventions to address public health problems	This course addresses how public health professionals take action to solve public health problems. Building on the foundation of understanding problems from PUB 320, the course addresses interventions used to prevent and treat infectious diseases, to change health behaviors, and to address environmental health hazards. A particular focus will be on intervention strategies that can be used at the population level to improve health for groups and communities.
PUB 330: Public health systems and policies	This course addresses how the public health system and the broader health care system function to promote health and treat illness, as well as how governments function to address public health issues. Major topics addressed will include the structure and function of the public health system in the United States, how those functions are provided for by law and financed by governments; the structure of the health care delivery system and how it relates to the public health system; policy design and implementation and the role of government in that design.
PUB 422: Public health ethics: an interdisciplinary exploration	Public Health Ethics explores interdisciplinary perspectives using literary, philosophical, and historical examples. Public health ethics has a special concern about functions of the state and organizations in protecting and promoting health. The American Public Health Association Principles of Ethical Practice of Public Health will be employed to assess important moral dilemmas presented in cases, literature, and films. Principles of moral philosophy and moral psychology will also be used.
PUB 494: Capstone: modern public health problems and solutions	This course satisfies the capstone requirement for the major in public health. The course focuses on integrating and synthesizing knowledge gained in the public health major core curriculum and using that knowledge to analyze, explain, and address public health problems. Students will also gain exposure to how knowledge from the core curriculum is applied in public health practice. The course will center around student projects based on case studies of public health problems.

### BSPH Curriculum—Distinctive Design Features

There are several advantages of using an integrative curriculum approach to the design and implementation of a BSPH program. First, the deliberate, distinctive design features of our UGPH curriculum allow for the careful and thoughtful integration of the five core public health areas in each and every one of the core courses throughout the curriculum, allowing for the high impact delivery of integrative learning experiences throughout the students' time in the major (36). In particular, students are presented with various opportunities for both breadth of exposure to core public content and ways of thinking and depth in a focus area of interest. The inclusion of a flexible capstone course allow synthesis and integration based on student experiences (e.g., undergraduate research, study abroad, internship), incorporating Kuh's capstone experiences as a high impact practice (37). Second, the leveraging of general education provides disciplinary foundations for public health learning at lower levels of the required curriculum. In designing the core courses, we identified general education offerings that provided important background knowledge relevant to each course and made those general education offerings pre-requisites to the public health core. For example, a human physiology general education course is a pre-requisite for the core course addressing biological, psychosocial, and environmental mechanisms of health and disease and a political science introductory course is a pre-requisite for the course on public health systems and policies. This leveraging of general education not only makes the curriculum stronger, but also illustrates for students the integration of core knowledge from multiple disciplines into the public health approach to addressing and understanding health. The University at Buffalo's core general education curriculum explicitly incorporates several high impact practices into its design, including first year experiences, use of portfolios, capstone experiences at the end of the general education sequence, common intellectual experiences, and diversity/global learning (37). Third, the curriculum design allows for two primary options for student learning experiences: many students will assume a “stand alone” baccalaureate experience and others may opt for a seamless transition into a BSPH + MPH without unnecessary duplication for master's level coursework (in either a 3+2 or 4+2 track). The curriculum development and implementation plans were explicitly informed by and responsive to Framing the Future. The content coverage takes a thoughtful approach to integrate the course content, allowing students to make often implicit connections between courses explicit. Importantly, the UGPH curriculum successfully incorporates Critical Component Elements (see Table 1) from ASPPH's Framing the Future project (38, 39).

### BSPH Course Examples

To highlight samples of what an integrative approach to curriculum development and implementation looks like, we present two course examples in our BSPH program: PUB 315: Asking and Answering Scientific Questions in Public Health and PUB 320: Models and Mechanisms for Understanding Public Health.

In PUB 315, students engage in an overview of scientific methodology and evidence-based practice in public health where they learn about the epidemiological research methods used to collect data and the biostatistical methods used to evaluate that data in public health research and practice. They begin the course by learning about the importance of evidence-based practice for public health with an overview of the research process and the development of research questions. Students examine the types of empirical questions addressed in the discipline and the links between the types of questions and appropriate methods to collect, manage, and analyze public health data. These learning synergies provide students the opportunity to deliberately engage in understanding the interrelation between not only key public health concepts but also draw connections between conceptual commonalities in all five core areas of the discipline.

In PUB 320, students engage in course material to learn about how we understand and explain the causes of public health problems. Using active learning techniques, students gain an understanding of the complex causal mechanisms of different types of public health problems, including infectious diseases, chronic diseases, and environmental health hazards. These active learning strategies include working in groups to develop and apply multilevel explanations of public health problems and to develop knowledge through activities designed to compare and contrast the ways in which different models in public health reflect systems thinking principles. By first learning how to apply basic principles of model building to analyze and explain public health problems, they then explore the importance of identifying and understanding the relationship between and among causes within complex systems using various levels of the social ecological model and the epidemiologic triad. Using experiential learning strategies, students gain skills in describing and explaining factors that influence health-related behaviors using public health theories and environmental models. For example, students work to create the textbook through for the course, curating, and writing about core content knowledge in a way that involves experiential engagement in knowledge creation (40). Finally, students explore fundamental causes and use model applications to reduce health disparities at a population level using principles in the five core areas of public health.

## Conclusion

Integrative learning is an educational practice intended to produce high-quality, long-lasting learning experiences. Consistent with CEPH requirements for the undergraduate public health major and learning outcome recommendations in the Framing the Future Task Force report, an integrative approach to curriculum design and implementation derives from the interdisciplinary roots of the public health profession and the key notion that public health work is fundamentally integrated in nature. From a curriculum design perspective, it is critical to develop an educational program at the bachelor's level that helps students establish knowledge, problem-solving, and critical thinking skills for understanding and analyzing the complexities involved in public health phenomena as well as fostering proficiencies to effectively respond to these complex problems in the “real world” work of public health practice. These competencies focus on the utilization of interdisciplinary, multi-level approaches. An integrative curriculum for undergraduate public health students prepares them to enter the public health workforce with thoughtful, deliberate synthesis of key principles in our field and provides them with disciplinary foundations for their future careers as public health practitioners and researchers.

## Author Contributions

MK contributed to the conceptualization and design of the manuscript. MK and SP co-wrote the first draft of the manuscript. Each author contributed to manuscript revision, read, and approved the submitted version.

### Conflict of Interest Statement

The authors declare that the research was conducted in the absence of any commercial or financial relationships that could be construed as a potential conflict of interest.

## References

[1] Kiviniemi MT Mackenzie SLC. Framing undergraduate public health education as liberal education: who are we training our students to be and how do we do that? Front Public Health. (2017) 5:9. 10.3389/fpubh.2017.00009 28239603 PMC5301016

[2] Gordis L Epidemiology. Philadelphia, PA: Saunders (2013).

[3] DeSalvo KB O'Carroll PW Koo D Auerbach JM Monroe JA. Public health 3.0: time for an upgrade. Am J Public Health. (2016) 106:621–2. 10.2105/AJPH.2016.303063 26959263 PMC4816012

[4] Rudolph L Caplan J Ben-Moshe K Dillon L Health in all Policies: A Guide for State and Local Governments. Washington, DC: American Public Health Association (2013).

[5] Zinsstag J Schelling E Waltner-Toews D Tanner M. From “one medicine” to “one health” and systemic approaches to health and well-being. Prev Vet Med. (2011) 101:148–56. 10.1016/j.prevetmed.2010.07.003 20832879 PMC3145159

[6] Jacobs HH Interdisciplinary Curriculum: Design and Implementation. Arlington, VA: Association for Supervision, Curriculum Development (1989).

[7] Meeth LR Interdisciplinary studies: a matter of definition. Change. (1978) 10:10 10.1080/00091383.1978.10569474

[8] Harden RM. The integration ladder: a tool for curriculum planning and evaluation. Med Educ. (2000) 34:551–7. 10.1046/j.1365-2923.2000.00697.x 10886638

[9] Fogarty R How to Integrate The Curricula. 3rd ed Thousand Oaks, CA: Sage (2009).

[10] Schmidt H. Integrating the teaching of basic sciences, clinical sciences, and biopsychosocial issues. Acad Med. (1998) 73(Suppl. 9):S24–31. 10.1097/00001888-199809001-00006 9759115

[11] Wykoff R Khoury A Stoots JM Pack R. Undergraduate training in public health should prepare graduates for the workforce. Front Public Health. (2015) 2:285. 10.3389/fpubh.2014.00285 25601906 PMC4283444

[12] Martin BC Stoots JM Pack RP Wykoff R Dreyzehner JJ. Potential approaches to address the undergraduate public health training needs for working professionals: a case study of one rural area. J Public Health Man. (2010) 16:128–33. 10.1097/PHH.0b013e3181c8cb37 20150794

[13] Rozier M Scharff D. The value of liberal arts and practice in an undergraduate public health curriculum. Public Health Rep. (2013) 128:416–21. 10.1177/003335491312800515 23997293 PMC3743295

[14] Council on Education in Public Health. Accreditation Criteria for Schools of Public Health & Public Health Programs. Washington, DC: CEPH (2016).

[15] Barr RB Tagg J From teaching to learning—A new paradigm for undergraduate education. Change. (1995) 27:12–26.

[16] Fink LD Creating Significant Learning Experiences. San Francisco, CA: Jossey-Bass (2003).

[17] Johnson S The Ghost Map: The Story of London's Most Terrifying Epidemic–and How it Changed Science, Cities, and The Modern World. New York, NY: Penguin (2006).

[18] Ambrose SA Bridges MW DiPietro M Lovett MC Norman MK How Learning Works: Seven Research-Based Principles for Smart Teaching. San Francisco, CA: Jossey-Bass (2010).

[19] Dewar DM Bloom MS Choi H Gensburg L Hosler A. The integrated first year experience in the master of public health program. Am J Public Health. (2015) 105(Suppl. 1):S97–8. 10.2105/AJPH.2015.302558 25706030 PMC4339982

[20] Cullen R Harris M Hill RR The Learner Centered Curriculum: Design and Implementation. San Francisco, CA: Jossey-Bass (2012).

[21] Wiggins G McTighe J Understanding by Design. 2nd ed Upper Saddle River, NJ: Pearson (2005).

[22] Harden RM Sowden S Dunn WR. Educational strategies in curriculum development: the SPICES model. Med Educ. (1984) 18:284–97. 10.1111/j.1365-2923.1984.tb01024.x 6738402

[23] Malik AS Malik RH. Twelve tips for developing an integrated curriculum. Med Teach. (2011) 33:99–104. 10.3109/0142159X.2010.507711 20874013

[24] Schmidt HG Machiels-Bongaerts M Hermans H ten Cate TJ Venekamp R Boshuizen HP. The development of diagnostic competence: comparison of a problem-based, an integrated, and a conventional medical curriculum. Acad Med. (1996) 71:658–64. 9125924 10.1097/00001888-199606000-00021

[25] Halpern DF Hakel MD Applying the science of learning to the university and beyond. Change. (2003) 35:36 10.1080/00091380309604109

[26] Association of American Colleges & Universities College Learning for The New Global Century: A Report From The National Leadership Council for Liberal Education & America's Promise. Washington, DC: Association of American Colleges and Universities (2007).

[27] Reynolds C Patton J Leveraging The ePortfolio for Integrative Learning. Sterling, VA: Stylus (2014).

[28] Regehr G Norman GR. Issues in cognitive psychology: implications for professional education. Acad Med. (1996) 71:988–1001. 10.1097/00001888-199609000-00015 9125988

[29] Marzano RJ Pickering DJ Brandt RS Integrating instructional programs through dimensions of learning. Educ Leadersh. (1990) 47:17.

[30] Caine RN Caine G Understanding a brain-based approach to learning and teaching. Educ Leadersh. (1990) 48:66.

[31] Ramirez AG Save our Science: How to Inspire a New Generation of Scientists [e-book]: TED Conferences. (2013). Available online at: https://www.amazon.com/Save-Our-Science-Generation-Scientists-ebook/dp/B00B7B0G32/ref=sr_1_1?ie=UTF8&qid=1541710958&sr=8–1&keywords=save+our+science (accessed November 15, 2018).

[32] Shatzer JH. Instructional methods. Acad Med. (1998) 73(Suppl. 9):S38–45. 10.1097/00001888-199809000-00034 9759117

[33] McGrath EJ An integration of knowledge and experience. Change. (1978) 10:6–9.10304691

[34] Huber MT Hutchings P Integrative Learning: Mapping The Terrain. Wasington, DC: Association of American Colleges and Universities (2004).

[35] Vidic B Weitlauf HM. Horizontal and vertical integration of academic disciplines in the medical school curriculum. Clin Anat. (2002) 15:233–5. 10.1002/ca.10019 11948961

[36] Association of American Colleges and Universities Greater Expectations: A New Vision for Learning as a Nation Goes to College. Washington, DC: AACU (2002).

[37] Kuh GD High-Impact Edcuational Practices: What They Are, Who Has Access to Them, and Why They Matter. Washington, DC: Association of American Colleges and Universities (2008).

[38] Petersen DJ Weist EM. Framing the future by mastering the new public health. J Public Health Man. (2014) 20:371. 10.1097/PHH.0000000000000106 24813674 PMC4032212

[39] Wykoff R Petersen D Weist E. The recommended critical component elements of an undergraduate major in public health. Public Health Rep. (2013) 128:421–4. 10.1177/003335491312800516 23997294 PMC3743296

[40] University at Buffalo Students Create Their Own Open Educational Resource Textbook. (2019). Available online at: https://www.aspph.org/university-at-buffalo-students-create-their-own-open-educational-resource-textbook/ (accessed February 28, 2019).

